# Drugs of Abuse in HIV infection and neurotoxicity

**DOI:** 10.3389/fmicb.2015.00217

**Published:** 2015-03-24

**Authors:** Melissa Hidalgo, Venkata S. R. Atluri, Madhavan Nair

**Affiliations:** Department of Immunology, Institute of NeuroImmune Pharmacology, Herbert Wertheim College of Medicine, Florida International UniversityMiami, FL, USA

**Keywords:** HIV, neurocognitive disorders, nicotine, methamphetamine, cocaine, cannabinoids, opioids, alcohol

## Introduction

**Figure 1:**
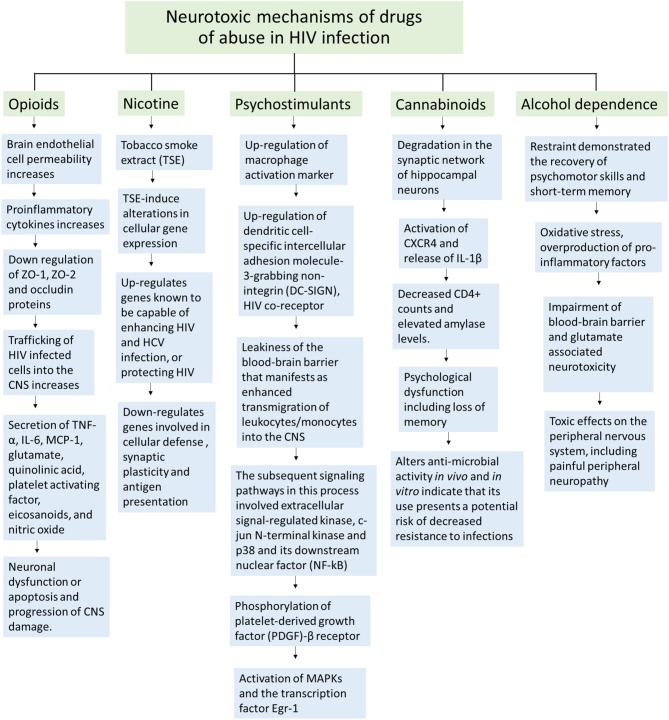
**Schematic representation of neurotoxic mechanisms of different illicit drugs of abuse in HIV infection**.

HIV is a neurotropic virus that enters the brain right after infection. In the brain, HIV replicates in macrophages, microglia and small number of astrocytes (4.7 ± 2.8% *in vitro* and 8.2 ± 3.9 *in vivo*) (Eugenin et al., 2011) causing inflammatory and neurotoxic host responses. Severe neurological disorders caused by HIV are collectively known as HIV-associated neurocognitive disorders (HAND). HAND is characterized by development of abnormal reduction of motor speed, concentration, and memory. HAND consists of several clinical forms ranging from asymptomatic neurocognitive impairment (ANI), minor neurocognitive disorder (MND) to the most severe HIV-associated dementia (HAD) (McArthur and Brew, 2010). HIV-encephalitis (HIVE) is the main cause of HAND and the most common neurologic disorder of the brain in HIV-1 infection. HIV-1 exhibits extensive genetic variation worldwide and is categorized into three groups (M, O, and N) and genetically into nine different subtypes (A–K). Of these, clades B and C represent the majority (>86%) of circulating HIV-1 variants (Osmanov et al., 2002). While HIV-1 clade B is predominant in North America, Western Europe, and Australia; clade C is common in Southern and East Africa, India and Nepal (responsible for around half of all HIV infections). HIV-1 clade B has been reported to be more neuropathogenic than clade C (Atluri et al., 2013; Samikkannu et al., 2014). Before the worldwide use of highly antiretroviral therapy (HAART), approximately, 20–30% of individuals with advanced HIV-1 clade B infection showed symptoms of HAD (Gonzalez-Scarano and Martin-Garcia, 2005; Kaul et al., 2005). Although the prevalence of HAD has decreased intensely after the introduction of HAART, 40–50% of HIV positive patients still suffer from HAND (Sacktor et al., 2001; Sacktor, 2002; McArthur, 2004; Antinori et al., 2007; Ellis et al., 2007). In developed countries, about 30% of HIV-positive individuals are intravenous drug abusers, which place them in a higher risk for HAND (Miro et al., 2003; Beyrer et al., 2010). Cocaine and marijuana are the most common drugs of abuse among HIV patients, whereas opioids are abused only by a small number of patients (Kuo et al., 2004; Cook et al., 2007; Korthuis et al., 2008). Overall, several drugs of abuse such as tobacco, stimulants, cannabinoids, opioids and alcohol are found to be consumed among HIV infected individuals, having an effect on synaptic plasticity and development found in the brain (Hauser and Knapp, 2014). Figure 1 is showing different neurotoxic mechanisms of drugs of abuse in HIV infection which may lead to the impaired neurocognitive functions.

## Nicotine and HIV

Recently, in nicotine and HIV infected SK-N-MC cells, an up-regulation of *HDAC2* was observed (Atluri et al., 2014). HDAC2 overexpression has been reported in depleted memory formation, synaptic plasticity and dendritic spine density (Guan et al., 2009). Nevertheless, use of nicotine in infected patients can have beneficial outcomes on neurological deficits that were HIV-1 induced (Cao et al., 2013). In nicotine injected HIV-1 transgenic rats brain regions, such as in the prefrontal cortex, Wnt/β-catenin signaling has shown improvement by restoring the down-regulation of *Axin1, Wnt5a, Wnt7a* and the up-regulation of *Gnao1* (Cao et al., 2013). This signaling pathway plays an important role in the early development of the nervous system, in which a neuroprotective outcome in adults is established after the activation of this pathway (Nave and Trapp, 2008). These results are important since central demyelination and neurodegeneration have been observed in HIV-1 infected individuals. In the dorsal hippocampus, CREB signaling has also been restored, in which the decreased expression of calcium sensor proteins, Calm3 and Cabp1, was regulated to normal levels. This signaling pathway is significant in neuronal survival and long-term synaptic plasticity. In the dorsal striatum, nicotine has also shown to restore the function of the tricarboxylic acid (TCA) cycle and its related pathways, such as the down-regulation of *Idh3B* and up-regulation of *Ndufs4* that came back to normal levels (Cao et al., 2013).

## Psychostimulants and HIV

Stimulants such as cocaine indicated to be involved in up-regulation of macrophage activation marker human leukocyte antigen (HLA)-DR and increased HIV replication in monocytes, and even astrocytes *in vitro* (Dhillon et al., 2007). Additionally, cocaine can induce HIV infection by the up-regulation of dendritic cell-specific intercellular adhesion molecule-3-grabbing non-integrin (DC-SIGN), which is another HIV co-receptor (Nair et al., 2004). Cocaine can also induce monocyte transendothelial migration, expression of endothelial adhesion molecules, and disruption of intercellular junctions in the blood brain barrier (BBB) *in vitro* (Fiala et al., 2005). HIV patients with cocaine dependence reported to have a greater neurocognitive impairment and poorer HAART adherence compared to non-drug users (Meade et al., 2011). Dendritic varicosity formation was enhanced in primary hippocampus neurons in the presence of HIV-1 envelope glycoprotein gp120 and cocaine (Yao et al., 2009). These findings proof that cocaine intensifies HIV infection and neuronal damage.

On the other hand, methamphetamine (meth) is a neurotoxic drug that injures dopaminergic neurons and reduces dopamine as well as norepinephrine in the brain (McCann et al., 1998). Moreover, in HIV infected meth abusers, a more cognitive impairment was related to a smaller hippocampal volume (Berman et al., 2008). The expression of HIV co-receptors, CXCR4 and CCR5, and HIV replication in astrocytes were both increased in presence of meth (Nair et al., 2009). BBB dysfunction and an increase in the expression of pro-inflammatory cytokinesis were observed, which can lead to meth-neurotoxicity (Czub et al., 2001; Nath et al., 2002). Meth and gp120 cooperated to induce interleukin-6 (IL-6) and cytochrome P450 2E1 (*CYP2E1*) in astrocytes (Shah et al., 2013). *CYP2E1* generated free electrons and reactive oxygen species (ROS) through its reaction cycle. Further, caspase-3 activity increased, leading to DNA fragmentation and apoptosis in astrocytes (Shah et al., 2013). The expression of ZO-1, claudin-3, claudin-5, and *JAM-2* also decreased in combination of Meth and gp120 or Tat (Mahajan et al., 2008; Banerjee et al., 2010). Levels of matrix metalloproteinase 2 (MMP-2) and MMP-9 secretions increased in the presence of meth and Tat treatment (Conant et al., 2004). These data then proofs exacerbation of neurotoxicity in HIV and meth combination.

## Cannabinoids and HIV

The consumption of cannabinoids, in HIV infected individuals was reported to modify inflammatory and neurotoxic processes. Studies on HIV gp120 treated cells demonstrated degradation in the synaptic network of hippocampal neurons (Kim et al., 2011). These occurred when activation of CXCR4, triggered cell signaling pathways and the release of IL-1β (Yi et al., 2004). This β-chemokine is responsible for mediation of synapse loss, which activates an ubiquitin ligase and the N-methyl-D-aspartate (NMDA) receptor. This receptor is responsible for the control of memory function and synaptic plasticity. The synapse loss was said to be a protective mechanism rather than an agonal event, which prevents cells from being over stimulated (Kim et al., 2011).

However, the cannabinoid receptor full agonist (Win55212-2) functioned as a protector for hippocampal neurons against gp120-induced IL-1β production and synapse loss. This protection has been reported to be mediated by the CB2 cannabinoid receptor, not the CB1. However, in exposure to the HIV-1 protein Tat, Win55212-2 did not inhibit synaptic loss (Kim et al., 2011). It was also reported that the cannabinoid CB2 receptor agonist (AM1241) intensifies neurogenesis *in vivo* and reduces astrogenesis and gliogenesis in the hippocampus of GFAP/Gp120 transgenic mouse model (Avraham et al., 2014). This is an important research content in the medical field for the future treatment of neurodegenerative diseases and related disorders.

Moreover, the consumption of marijuana and its effects on cognitive function is controversial. Marijuana consumption is a common practice in HIV-infected individuals for different interests, such as recreational and medicinal purposes. However, reports are scanty on the impact of marijuana on cognitive functions in these subjects. In a cohort study, the measurement of cognitive impairment demonstrated synergistic effects of marijuana consumption in individuals with advanced HIV disease whereas minimal impact on uninfected or those at early stages of HIV infection (Cristiani et al., 2004).

## Opioids and HIV

HIV viral proteins and opioid drugs are known as cofactors for HIV infection because they act in synergy, leading to a greater immunosuppression. HIV-1 Tat and morphine demonstrated a synergistic effect in the up-regulation of inflammatory cytokines and chemokines as well as a reduction in total spine density (Fitting et al., 2011). This was observed when μ-opioid receptor (MOPr) interacted with glutamatergic signals that came from α-amino-3-hydroxy-5-methyl-4-isoxazolepropionic acid (AMPA) receptors (Liao et al., 2005). The over activation of NMDA receptor by Tat (Li et al., 2008), caused an excitotoxic manifestation that inhibited ATP production and disruption in cellular energetics (Perry et al., 2005). Fitting et al. reported a novel finding on HIV-1 Tat and morphine-induced synaptodendritic injury mediated by increased intracellular sodium ([Na(+)]i) and calcium ([Ca(2+)]i) in dendrites (Fitting et al., 2014).

In addition, the disturbance of synaptic transmissions in dorsolateral prefrontal cortex (DLPFC) has been observed in subjects that exhibit HIV encephalitis (HIVE) or HIV-associated neurocognitive disorders (HAND). In HIVE patients, expression of preproenkephalin (PENK), an opioid neurotransmitter, was reduced and interferon regulatory factor 1 (IRF1) expression was increased (Gelman et al., 2012); these are related neuropathologically to HIVE.

## Alcohol dependence and HIV infection

It has been reported that lifetime alcohol dependence is able to impair memory, attention and learning. However, cognitive dysfunctions are not all transient (Darke et al., 2000). Although restraint demonstrated the recovery of psychomotor skills and short-term memory; long-term memory injuries were observed to last up to 7 years (Brandt et al., 1983). In a comparative study between HIV-infected and uninfected individuals that had an alcohol dependence history, major cognitive impairment in HIV-infected group was observed within the domains of reaction time, auditory processing, and verbal reasoning, whereas no major impairments were observed in the other group. Thus, this showed the synergistic effects that HIV and alcohol have on the central nervous system (CNS) (Green et al., 2004).

## Clinical studies on effect of drugs of abuse in HIV associated neurocognitive impairment

Several studies have associated cocaine, methamphetamine and opioid use with the aggravation of the risk for neurocognitive impairment and neuronal injury in HIV infected individuals (Martin-Thormeyer and Paul, 2009; Banerjee et al., 2011; Byrd et al., 2011; Dutta and Roy, 2012). Nonetheless, a recent large cohort study found that participants with histories of substance use (alcohol, cocaine, cannabis, opiates, and methamphetamine) did not exhibit higher rates of neurocognitive or functional impairment in everyday life (Byrd et al., 2011). The majority of these individuals were not current users and less than a third reported using illicit substances within the last year. According to the authors, continuous phases of drug abstinence might be enough for a full or partial recovery from neurocognitive impairment. These results are consistent with another longitudinal study, in which a neurocognitive function improvement was observed in long-term abstinent meth users (average of 13 months) in comparison with non-abstinent meth users (Iudicello et al., 2010). These findings propose that drugs of abuse might have a limited legacy outcome on neurocognitive injury in HIV infected individuals and that recent substance use may be more relevant to modulating HIV neuropathogenesis. Further supporting a role for drugs of abuse increasing neuroinflammation and associated neurodegeneration, HIV-encephalitis diagnosis is more frequent in HIV positive individuals who abuse drugs in comparison to HIV positive controls (Buch et al., 2012; Liu et al., 2012).

## Conclusions

An understanding of the close and bidirectional relationship between cognitive impairment and HIV risk factors is essential to efficiently reduce HIV risk. Clearly, the use of drugs of abuse plays a critical role in HIV infected individuals, affecting their neurological functioning and pathogenesis of HIV. Loss of synaptic connections has been associated with HIV-1 associated dementia (HAD). However, since there are inhibitory and excitatory connections, then this synapse loss, induced by HIV-1, Tat, gp120 proteins in association with drugs of abuse, is reversible. These data contribute to the improvement of neuropathological outcomes in HIV individuals by offering new insights for possible medical treatments that will target inhibitory synapses among other techniques to treat HAD, HIVE, HAND and related neurodegenerative diseases in drug abusing patients. Further studies exploring the specific effects of applying cognitive remediation approaches in HIV-specific interventions should be directed.

### Conflict of interest statement

The authors declare that the research was conducted in the absence of any commercial or financial relationships that could be construed as a potential conflict of interest.
